# Early versus late anticoagulation for ischaemic stroke associated with atrial fibrillation: multicentre cohort study

**DOI:** 10.1136/jnnp-2018-318890

**Published:** 2018-11-19

**Authors:** Duncan Wilson, Gareth Ambler, Gargi Banerjee, Clare Shakeshaft, Hannah Cohen, Tarek A Yousry, Rustam Al-Shahi Salman, Gregory Y H Lip, Henry Houlden, Martin M Brown, Keith W Muir, Hans Rolf Jäger, David J Werring

**Affiliations:** 1 Department of Brain Repair and Rehabilitation, UCL Queen Square Institute of Neurology and the National Hospital for Neurology and Neurosurgery UCL Stroke Research Centre, London, UK; 2 Department of Statistical Science, University College London, London, UK; 3 Haemostasis Research Unit, Department of Haematology, University College London, London, UK; 4 Lysholm Department of Neuroradiology and the Neuroradiological Academic Unit, Department of Brain Repair and Rehabilitation, UCL Queen Square Institute of Neurology, London, UK; 5 Centre for Clinical Brain Sciences, School of Clinical Sciences, University of Edinburgh, Edinburgh, UK; 6 Institute of Cardiovascular Sciences, University of Birmingham, Birmingham, UK; 7 Department of Molecular Neuroscience, UCL Queen Square Institute of Neurology and the National Hospital for Neurology and Neurosurgery, London, UK; 8 Institute of Neuroscience and Psychology, University of Glasgow, Queen Elizabeth University Hospital, Glasgow, UK

**Keywords:** ischaemic stroke, anticoagulation, warfarin, DOAC, atrial fibrillation

## Abstract

**Background and purpose:**

The optimal time to start oral anticoagulant (OAC) in patients with ischaemic stroke due to non-valvular atrial fibrillation (AF) is unknown. We reviewed OAC timing in relation to 90-day clinical outcomes as a post hoc analysis from a prospective multicentre observational study.

**Methods:**

We included patients with data on time to initiation of OAC from CROMIS-2 (Clinical Relevence Of Microbleeds In Stroke-2), a prospective observational inception cohort study of 1490 patients with ischaemic stroke or transient ischaemic attack (TIA) and AF treated with OAC. The primary outcome was the composite outcome of TIA, stroke (ischaemic stroke or intracranial haemorrhage) or death within 90 days of the qualifying stroke or TIA. We performed adjusted logistic regression analyses to compare early (0–4 days) and later (≥5 days or never started) OAC initiation.

**Results:**

We included 1355 patients, mean age 76 (SD 10), 580 (43%) women. OAC was started early in 358 (26%) patients and later (or not at all) in 997 (74%) patients. The event rate within 90 days was 48/997 (5%) in the late-OAC group (2 intracranial haemorrhages, 18 ischaemic strokes or TIAs and 31 deaths (three deaths were as a result of new ischaemic strokes)) versus 7/358 (2%) in the early-OAC group (5 ischaemic strokes or TIAs and 2 deaths). In adjusted analyses, late OAC was not associated with the composite outcome (adjusted OR 1.17, 95% CI 0.48 to 2.84, p=0.736).

**Conclusion:**

In adjusted analyses, early OAC after acute ischaemic stroke or TIA associated with AF was not associated with a difference in the rate of the composite outcome of stroke, TIA or death at 90 days, compared with late OAC. However, despite adjustment for important baseline factors, patients selected for early OAC and late OAC might still have differed in important respects; evaluation of OAC timing in adequately powered randomised trials is required.

**Clinical trial registration:**

NCT02513316.

## Introduction

Oral anticoagulation (OAC) is recommended for the secondary prevention of ischaemic stroke due to non-valvular atrial fibrillation (AF),^1 2^ but the optimal timing of OAC following an acute ischaemic stroke or transient ischaemic attack (TIA) is unknown.^3 4^ In AF-related acute ischaemic stroke, the risk of early recurrence (within 7–14 days) is between 0.4% and 1.3% per day.^5–9^ AF-related ischaemic strokes are more often disabling or fatal than other types, with longer hospital stays and higher costs,^10^ so preventing early recurrence is a key clinical challenge. OAC is highly effective for long-term stroke prevention in AF,^11^ but the safety and net benefit in acute AF-related stroke have not been established.

Initiation of OAC in the first few days after stroke could prevent ischaemic stroke recurrence but might increase the risk of symptomatic intracranial haemorrhage (ICH), including haemorrhagic transformation of the infarct (estimated at ~9% in the first 7 days),^12^ leading to clinical uncertainty about when to start OAC. Recent observational studies (mostly including patients treated with warfarin or other vitamin K antagonists) reported an 8%–10% risk of recurrent ischaemic stroke and a 2%–4% risk of symptomatic ICH within 90 days of AF-related ischaemic stroke.^13 14^ Current guidelines do not provide clear recommendations on the timing of OAC after acute AF-related stroke: US guidelines suggest that commencing OAC within 14 days is reasonable.^15^ The 14-day time delay is likely in part derived from the European Atrial Fibrillation Trial where the time to anticoagulation was within 14 days in only 43% of patients,^16^ as well as observational CT-based data suggesting that most haemorrhagic transformation occurs within 14 days of an ischaemic stroke.^17^ The European Society of Cardiology guidelines recommend starting OAC according to infarct size at 1, 3, 6 or 12 days based only on expert consensus.^18^ Current UK guidelines for OAC state that ‘delay for an arbitrary 2 week period is recommended’ for ‘disabling’ stroke and that OAC can be started ‘no later than 14 days’ for other strokes, at the prescriber’s discretion (see https://www.strokeaudit.org). Indeed, recent trials of non-vitamin K oral anticoagulants (direct oral anticoagulants, DOACs) excluded patients who were started on a DOAC within 7–14, and up to 30, days of their ischaemic event.^19–21^

There have been a number of recent retrospective and observational studies exploring early OAC use in acute stroke (especially with DOACs) suggesting that early OAC is not associated with high rates of stroke events or death,^13 14 22–26^ and there are ongoing randomised control trials which aim to explore the safety and efficacy of early OAC in ischaemic stroke.^27–29^

In the present study, we describe the timing of OAC use in a prospective multicentre inception cohort observational study of adult patients presenting with acute ischaemic stroke or TIA and either known or new-onset AF, for which OAC was to be started. We reviewed the clinical and radiological characteristics of patients treated with early (0 to 4 days) versus late (5 to 90 days) OAC initiation, and their subsequent clinical outcomes.

## Materials and methods

Participants were recruited as part of the Clinical Relevence Of Microbleeds In Stroke-2 study (CROMIS-2), the protocol for which has been described elsewhere,^30^ but briefly CROMIS-2 is a multicentre, prospective inception observational study investigating whether cerebral microbleeds and other imaging markers of small vessel disease increase the risk of subsequent intracranial haemorrhage in patients with recent stroke and concurrent AF treated with anticoagulation. Patients were recruited from 79 hospitals throughout the UK and one hospital in the Netherlands. Patients were included if they presented with an ischaemic stroke or TIA, had AF and were judged by their treating physician as suitable for OAC. Patients were excluded if they had previously been exposed to OAC. Patients were included in this substudy if accurate information on the timing of OAC administration was available. The primary outcome was defined as a composite first of ischaemic stroke, TIA, ICH or death due to any cause within 90 days of the qualifying ischaemic event. Patients who had multiple events were not double counted when considering our composite event. Subsequent analyses were then performed individually for ischaemic events (ischaemic stroke and TIA), ICH and mortality. All stroke events required a clinical history suggestive of a vascular event with supporting neuroradiology and were independently adjudicated by DW (Clinical Research Fellow) and DJW (Professor of Clinical Neurology) blinded to timing of OAC.

Timing of OAC following the index ischaemic event was dichotomised into early (4 days or earlier) and late (5 days and onwards or anticoagulation never started) based on evidence showing that early (≤4 days) might be safe.^31^ Patients in which the exact date of anticoagulation was unknown were excluded from the analysis.

Baseline clinical and demographic variables and imaging findings relating to the qualifying event were visually compared between the early and late groups. Groups were compared using the Mann-Whitney U test if not normally distributed and the t-test if normally distributed; categorical variables between the groups were compared with the χ^2^ test or, when appropriate, Fisher’s exact test. Imaging studies from the qualifying stroke were rated for whether the index lesion fulfilled criteria for small vessel occlusion (defined as single small <15 mm Diffusion Weighted Imaging (DWI) infarctions within the middle cerebral artery distribution or the pons) and for infarct size (defined as greater or less than one-third of a vascular territory). Imaging was also rated for haemorrhagic transformation using the European Cooperative Acute Stroke Study (ECASS) criteria.^32^ Cerebral microbleeds and white matter hyperintensities (WMHs) were rated using validated scales.^33 34^ WMHs were rated using the Fazekas scale; a score of 2 or above in either a periventricular or deep white matter region was considered moderate to severe.

We undertook bivariable analyses of our baseline variables to decide whether to include each variable in the multivariable model. We first fitted the logistic regression model with anticoagulant timing as the only variable in the model and recorded the estimated regression coefficient. Then, one at a time, we adjusted for potential confounder variables and recorded whether there was a ‘clinically significant’ change (defined as a 10% absolute change or more) in the coefficient for timing. If there was, this variable was selected for the multivariable model. A multivariable logistic regression model was undertaken comparing early versus late anticoagulation for our composite outcome and each component of the composite outcome. ORs are interpreted as risk ratios as the outcome was relatively rare.

Sensitivity analyses were also completed in order to compare those with early (0 to 4 days), later (5 to 14 days) and late (15 days or later or not started at all) OAC start timings. Lastly, an exploratory analysis was undertaken excluding patients with TIAs and minor stroke (defined as National Institute of Health Stroke Score (NIHSS) <2) in whom early OAC is recommended.

## Results

**Figure 1:**
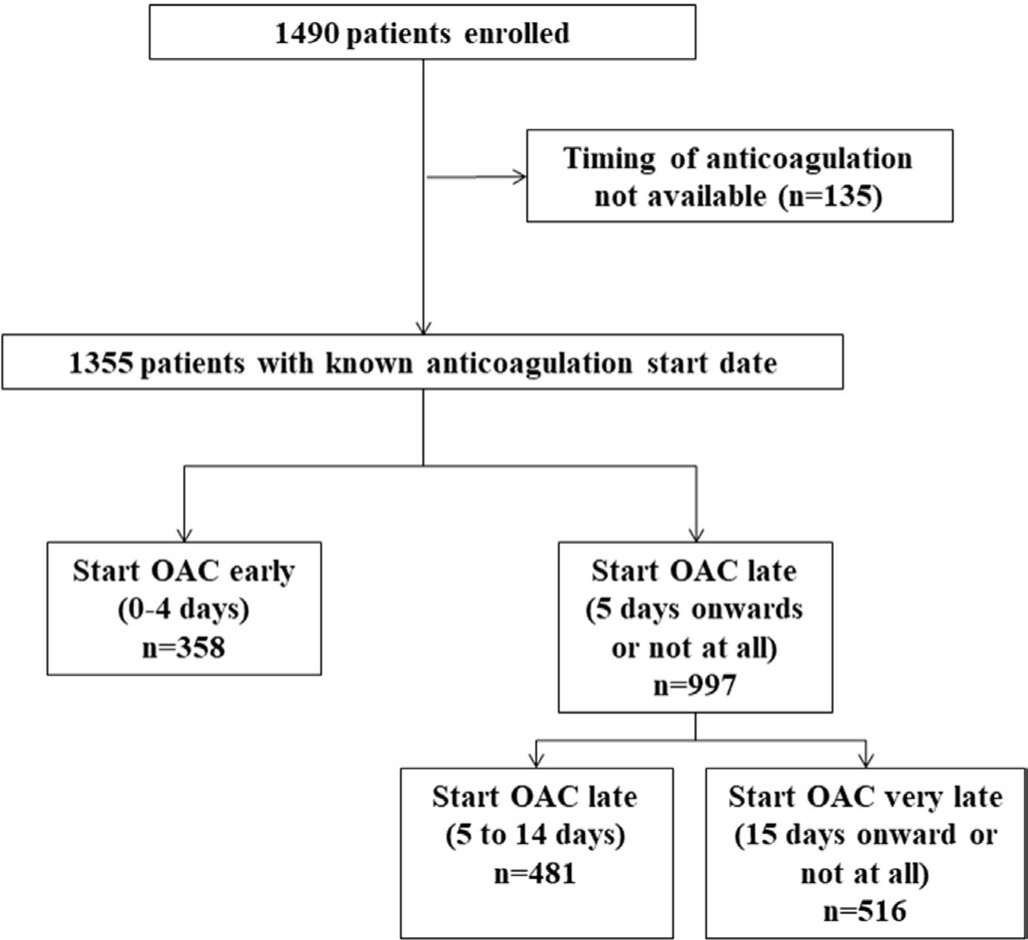
Flow chart of patient inclusion. OAC, oral anticoagulant.

There were 1355 patients with a known OAC start date (figure 1), of whom only 54 did not start an OAC. Our cohort was similar in baseline demographics and risk factor profile to patients in whom the timing of anticoagulation initiation was not known (n=135) (data not shown). The mean age was 76 years (SD 10) and 570 (43%) were women. The median NIHSS was 4 (IQR 2–10). A total of 235 (18%) patients had infarcts involving more than one-third of a vascular territory while 98 (8%) had lesions consistent with small vessel occlusion (table 1). The median time to OAC was 11 days (IQR 4–17); 839 (62%) had been started on OAC by day 15. Three hundred fifty-eight patients (26%) were started on OAC 4 days or earlier after their index stroke. Four hundred seventy-five patients (35%) were started on DOACs, and the remainder were started on warfarin.

**Table 1:** Demographics and risk factors between those anticoagulated early and late

Variable	All (n=1355)	Anticoagulated early (n=358)	Anticoagulated late or not at all (n=997)
Age, mean (SD)	76 (10)	75 (11)	76 (10)
Sex, female, n (%)	580 (43)	147 (41)	433 (43)
Hypertension, n (%)	846 (63)	211 (59)	635 (65)
Diabetes mellitus, n (%)	222 (16)	54 (15)	168 (17)
Hyperlipidaemia, n (%)	597 (45)	156 (44)	441 (45)
NIHSS, median (IQR)*	4 (2–10)	2 (1–4)	6 (3–11)
Previous stroke/TIA, n (%)	277 (21)	77 (22)	200 (20)
Previous ICH, n (%)	13 (1.0)	1 (0.28)	12 (1.22)
Antiplatelet therapy prior to qualifying stroke, n (%)	703 (52)	198 (55)	505 (51)
Known cognitive impairment or dementia, n (%)	32 (3)	9 (3)	23 (3)
Premorbid mRS, median (IQR)	1 (1–3)	1 (0–2)	2 (1–3)
Dependent living prior to stroke, n (%)	144 (12)	12 (4)	132 (16)
Thrombolysis, n (%)	263 (19)	43 (12)	220 (22)
Bridging heparin, n (%)	324 (24)	93 (26)	231 (23)
CHA_2_DS_2_-VASc score, median (IQR)	5 (4–6)	5 (4–6)	5 (4–6)
Qualifying ischaemic stroke >1/3 territory, n (%)	242 (18)	22 (6)	220 (22)
Qualifying ischaemic stroke lacunar, n (%)	98 (8)	28 (8)	70 (8)
PH1 Haemorrhagic transformation, n (%)	17 (1.3)	0 (0%)	17 (1.8)
DOAC on discharge, n (%)	475 (37)	164 (46)	311 (34)
Statin on discharge, n (%)	1043 (79)	266 (75)	777 (81)
Antiplatelet on discharge	717 (53)	114 (32)	603 (60)
Cerebral microbleed presence, n (%)	269 (21)	74 (21)	195 (21)
Moderate to severe WMH, n (%)	368 (29)	93 (26)	275 (30)

*Available in 851 of 1080 (79%) of patients with non-minor ischaemic stroke.

DOAC, direct oral anticoagulant; ICH, intracranial haemorrhage; mRS, modified Rankin Scale; NIHSS, National Institute of Health Stroke Score; PH1, parenchymal haemorrhage type 1; TIA, transient ischaemic attack; WMH, white matter hyperintensity.

Comparison of baseline characteristics and risk factors (table 1) showed that those who were started early were more likely to have been started on a DOAC (46% vs 35%) and less likely to have received intravenous thrombolysis (12% vs 22%), have a large infarct (6% vs 22% involving more than one-third of a vascular territory) or have significant haemorrhagic transformation (defined as parenchymal haemorrhage type 1 (PH1) or above) of the index infarct on MRI (0% vs 1.8%). Those anticoagulated early also had less severe strokes (median NHISS 2 vs 6) and lower pre-stroke functional disability (pre-stroke modified Rankin Scale (mRS) 1 vs 2), less dependent living (mRS >3) (4% vs 16%) and less likely to have had a previous ICH (0.28 vs 1.22%). Imaging markers of small vessel disease and previous antiplatelet use prior to index stroke were similar between the early and late OAC groups (55% vs 51%). However, patients who started OAC late were more likely to be on antiplatelet therapy at discharge from hospital (60% vs 32%) and at 6 months (although outside of the study follow-up window) (11% vs 4%, compared with those who started OAC early). Statin use at discharge was similar between the early and late groups (75% and 81%, respectively).

Ninety-day follow-up was available in all patients. Overall, 55 patients had at least one event within 90 days (event rate 4.1%). The median time to ischaemic stroke was 14 days (IQR 7–39) whereas the median time to ICH was 72 days (IQR 62–82). The event rate was 48/997 (5%) in the late-OAC group (two ICHs, 16 ischaemic strokes (all cardioembolic), two TIAs and 31 deaths (three deaths due to new ischaemic strokes)) versus 7/358 (2%) in the early-OAC group (three ischaemic strokes (all cardioembolic), two TIAs and two deaths). Of the 33 total deaths, 13 were vascular (three new ischaemic strokes, four myocardial infarctions, three instances of heart failure, one instance of gangrene and two patients who deteriorated after their original stroke), 11 were non-vascular and 9 were of unknown cause. In univariable analysis, patients starting OAC late were 2.5 times more likely to experience the composite outcome than patients who started OAC early (OR 2.54, 95% CI 1.14 to 5.66). Bivariable analysis showed that premorbid mRS, CHA_2_DS_2_VASC score and DOAC use were potential confounders, so these were included in multivariable analysis. After adjusting for all the potential confounders, multivariable analysis showed little evidence for a difference in composite outcome according to timing of OAC (late OAC OR 1.17, 95% CI 0.48 to 2.84) (table 2). Backwards stepwise regression revealed premorbid mRS (OR 1.48; 95% CI 1.16 to 1.90) to be the most strongly related with outcome and the major determinant of change in OR.

**Table 2:** Multivariable model showing the odds of the composite outcome (TIA, stroke or death) within 90 days

Variable	Units	OR (95% CI)	P values
Anticoagulant timing	Early	Reference	
	Late	1.17 (0.48 to 2.84)	0.736
Premorbid mRS	Per point	1.48 (1.16 to 1.90)	0.002
CHA_2_DS_2_VASC	Per point	1.21 (0.92 to 1.58)	0.169
DOAC medication on discharge	VKA	Reference	
	DOAC	1.29 (0.64 to 2.59)	0.475

DOAC, direct oral anticoagulant; mRS, modified Rankin Scale; TIA, transient ischaemic attack; VKA, vitamin K agonist.

Exploring components of the composite outcome in multivariable analysis (after running bivariable analysis for each outcome) showed no evidence of a difference between early and late OAC for risk of ischaemic stroke or TIA (OR starting late OAC 1.25, 95% CI 0.36 to 4.41) (table 3), or death (OR starting late OAC 0.91, 95% CI 0.20 to 4.60) (table 4). There was no evidence for a difference in risk of ICH (but with too few events to undertake logistic regression (0 vs 0.2%), Fisher’s exact test: p=1.00).

**Table 3:** Multivariable model showing the odds of recurrent ischaemic stroke or TIA within 90 days

Variable	Units	OR (95% CI)	P values
Anticoagulant timing	Early	Reference	0.727
	Late	1.25 (0.36 to 4.41)	
Intravenous thrombolysis	No	Reference	0.399
	Yes	0.48 (0.09 to 2.63)	
NIHSS	Per point	0.97 (0.85 to 1.11)	0.693
Premorbid mRS	Per point	0.84 (0.52 to 1.36)	0.470

Age, CHA_2_DS_2_VASC and infarction size were also idenfified as potential confounders. Due to the small number of events and a relative smaller effect size on the timing coefficient, these were not entered into the model to avoid overfitting.

NIHSS, National Institute of Health Stroke Scale; mRS, modified Rankin Scale.

**Table 4:** Multivariable model showing the odds of death within 90 days

Variable	Units	OR (95% CI)	P values
Anticoagulant timing	Early	Reference	
	Late	0.91 (0.20 to 4.60)	0.907
CHA_2_DS_2_VASC	Per point	1.14 (0.75 to 1.75)	0.544
Premorbid mRS	Per point	3.69 (2.22 to 6.14)	<0.001
DOAC medication on discharge	VKA	Reference	
	DOAC	1.42 (0.48 to 4.16)	0.527

DOAC, direct oral anticoagulant; VKA, vitamin K agonist.

Twenty-two out of 242 (9%) patients with large territorial infarctions were started on OAC early; none of these patients died or had an ICH within 90 days, but one had a recurrent ischaemic stroke. Haemorrhagic transformation was identified in 203 patients on their baseline MRI scans (which all included T2* Gradient Recalled Echo (GRE) blood-sensitive sequences). Using the ECASS criteria,^32^ 102 cases were HI1, 84 were HI2 and 17 were PH1. There were no instances of PH2. PH1 was more common in the later OAC group (0% vs 1.8%, p=0.005). There was no statistical difference between HI2 (36% vs 41% p=0.785) or HI1 (64% vs 50% p=0.388) between the early and late OAC groups

Multivariable sensitivity analyses comparing later (5 to 14 days) (n=481) and very late OAC (≥15 days or not started at all) (n=516) to early OAC (0 to 4 days) (n=358) show little difference in the odds of our primary composite outcome for these groups: starting late, OR 1.19(95% CI 0.45 to 3.90); starting very late, OR 1.14 (95% CI 0.42 to 3.09).

In an exploratory analysis of 1080 participants, excluding patients with TIA and minor stroke (n=275), 43 individual patients had 45 events within 90 days (event rate 4.2% within 90 days): 14 ischaemic strokes, 2 ICHs and 29 deaths. There was little evidence to suggest a difference in our primary composite outcome for the late and early OAC groups (late vs early OAC OR 0.72; 95% CI 0.19 to 2.73, p=0.630).

## Discussion

We show that current practice regarding timing of OAC in patients with stroke due to AF varies widely but broadly reflects the current guideline considerations and reasonable clinical judgement. Just over 25% of our patients were started on OAC by 4 days. Patients started on early OAC had milder strokes (less severe and of smaller size), were less likely to have been thrombolysed and had better pre-stroke functioning (as defined by mRS). In adjusted analyses, patients started on early OAC had similar odds for the composite end point of intracerebral haemorrhage, ischaemic stroke and death compared with those started later.

Our findings are consistent with other recent studies in which early OAC (variably defined as either <4 days,^31^ 4–14 days^13^ or <7 days^22^) was associated with low event rates of ischaemic stroke, ICH and death.^13 22 31^ While our event rate was much lower than those previously reported for the RAF study^13^ (12.6% at 90 days) and VISTA pooled trials analysis^14^ (13% at 90 days), it was similar to a DOAC cohort from Switzerland (7 events in 204 patients (3.4%); median time to event 28 days). The low rate of ischaemic stroke is perhaps in keeping with the expected rates extrapolated from the median CHA_2_DS_2_-VASc score, while the low ICH rate might be because of the high proportion of patients treated with DOACs, which are known to have about half the risk of ICH as VKAs.^2^ Similar to the Swiss cohort, CROMIS-2 selected patients who were MRI tolerant, an inclusion criteria not used in the RAF or VISTA trials, which may also go some way to explaining the lower event rates we found.

Our study confirms the wide UK variation in the timing of OAC administration after ischaemic stroke or TIA in patients with AF, possibly reflecting clinician uncertainty and ambiguity in available guidelines. In our study, of those anticoagulated, 34% were anticoagulated after day 14 and 17% after day 20. We did not collect information on why these patients had their OAC substantially delayed. Although PH1 was over-represented in patients anticoagulated late, it only accounted for 17 of 997 (2%) patients and so is unlikely to be the sole cause for the delay in OAC in the majority of these patients. It is possible that some of these delays were due to logistical reasons, for example as a consequence of communication or transfer of care at discharge, though we could not measure this. Starting OAC early (within 4 days) could therefore have an important practical advantage because the responsibility for initiating OAC would be taken by the acute stroke team, making logistical and communication challenges much less likely.

Our logistic models should be interpreted with caution and should not be used to guide clinical decisions. Death by 90 days was the main cause of difference in event rates between those starting OAC early versus those who started late in our composite outcome, and is nearly fully accounted for by the addition of premorbid mRS into a multivariable model. In this observational study, commencement of OAC was deferred in the face of more severe stroke and more severe comorbidity, so late OAC might be expected to be associated with higher mortality. Moreover, most of the deaths were due to non-vascular causes so might not be related to OAC timing. Our results, showing no evidence of hazard from early OAC, should nevertheless help inform future randomised control trials.

Strengths of our study include its multicentre design, making our results reflective of current UK practice. In our multivariable analysis, we adjusted for stroke severity and infarct size as well as CHA_2_DS_2_-VASc and estimated pre-stroke mRS, the major differences between the two groups. The main limitation of the study is its observational nature, where unmeasured treatment biases are impossible to adjust for, including clinician characteristics and patient frailty. Further markers of patient frailty, such as ‘do not rescuscitate’ orders, were not available. The low number of events within 90 days, specifically intracerebral events, precludes any further investigation into predictors of early haemorrhage. As stroke to MRI time was not standardised, more detained assessment of baseline infarct volume was not possible. A standardised MRI imaging time with assessment of DWI lesion volumes would be preferable.

Lastly, patients had to tolerate an MRI brain to be enrolled in CROMIS-2, which is likely to have led to the exclusion of older patients with more severe strokes, contributing to the median NIHSS of 4. Individuals with severe stroke, HF or unable to provide consent personally or via a consultee are likely to have a higher risk of events during the first 3 months, but were not included. Our findings are therefore only generalisable to patients with mild to moderately severe stroke who can undergo brain MRI.

Nevertheless, our data suggest that starting OAC early after ischaemic stroke is associated with low event rates of recurrent ischaemic stroke, ICH and death in selected patients, but the benefits and risks need to be tested in randomised trials over a full range of stroke severities. While we found a moderate (non-significant) effect size, the rarity of early events might require large sample sizes. We suggest randomised control trials should therefore consider similar designs and common clinical outcomes to allow for subsequent pooled individual patient data meta-analyses.
